# Development of the cartilaginous connecting apparatuses in the fetal sphenoid, with a focus on the alar process

**DOI:** 10.1371/journal.pone.0251068

**Published:** 2021-07-12

**Authors:** Masahito Yamamoto, Hiroaki Abe, Hidetomo Hirouchi, Masaki Sato, Gen Murakami, José Francisco Rodríguez-Vázquez, Shinichi Abe

**Affiliations:** 1 Department of Anatomy, Tokyo Dental College, Chiyoda-ku, Tokyo, Japan; 2 Department of Biology, Tokyo Dental College, Chiyoda-ku, Tokyo, Japan; 3 Division of Internal Medicine, Jikou-kai Clinic of Home Visits, Sapporo, Japan; 4 Faculty of Medicine, Department of Anatomy and Human Embryology, Institute of Embryology, Complutense University, Madrid, Spain; Laboratoire de Biologie du Développement de Villefranche-sur-Mer, FRANCE

## Abstract

The human fetal sphenoid is reported to have a cartilaginous connecting apparatus known as the alar process (AP), which connects the ala temporalis (AT) (angle of the greater wing of the sphenoid) to the basisphenoid (anlage of the sphenoid body). However, how the AP develops in humans is unclear. In addition, although the AP is a common structure of the mammalian chondrocranium, little is known about whether it is really a fundamental feature in mammals. This study examined the histological sections of 20 human embryos and fetuses from 6 to 14 weeks of development, of 20 mouse embryos from embryonic days 12–18, and of 4 rats embryos form embryonic days 17 and 20. In addition, we reconsidered the definition of the AP by comparing humans and rats with mice. In humans, the AP was continuous with the basisphenoid but was separated from the AT by a thick perichondrium. Then, the AP–AT connection had a key-and-keyhole structure. Unlike a joint, no cavitation developed in this connection. In mice, there was no boundary between the AT and the basisphenoid, indicating the absence of the AP in the mouse chondrocranium. In rats, the AP was, however, separated from the AT by a thick perichondrium. Therefore, the AP can be defined as follows: the AP is temporally separated from the AT by a thick perichondrium or a key-and-keyhole structure during the fetal period. This is the first study that confirms the absence of the alar process in the mice skull, and its presence in other mammals skull should be further investigated.

## Introduction

The sphenoid bone is a part of the skull, located in the middle part of the cranial base. It comprises a body, greater and lesser wings, and pterygoid processes and connects the neurocranium to the facial skeleton. The sphenoid has several foramina that allow the passage of many nerves and arteries [1]. It is formed through both endochondral and intramembranous ossification [2–6] and develops from distinct cartilaginous structures: the ala temporalis (AT), orbitosphenoid, presphenoid, and basisphenoid [7–9]. Its body is derived from the presphenoid and basisphenoid. Laterally, endochondral ossification centers appear in the AT for the greater wing (GW) and in the orbitosphenoid for the lesser wing [1].

The human fetal sphenoid has a cartilaginous connecting apparatus. Hannover [10] first documented a distinct bone between the cranial base and the AT and named it the “alar process (AP)”. In the human embryo, the AP is continuous with the basisphenoid at 7 weeks of development (WD) but is separated from the AT by a thick perichondrium [11]. Yamamoto et al. [12] showed that at 6 WD, the AP is a cell mass undistinguishable from the AT. They both develop into cartilaginous structures at 9 WD. Although human skull development is well studied [13], knowledge of AP development in the human sphenoid is incomplete.

The AP is a fundamental feature of the mammalian chondrocranium [7]. Veit [14] argued that the AP is located between the primary side wall and the cranial base of the skull and is homologous with the reptilian pterygoid process. Youssef [15] also identified the AP between the AT and the cranial base in albino rats. According to McBratney-Owen et al. [16], in C57BL6J mice, the AP is a basitrabecular process located between the AT and the cranial base. Although these studies have shown the AP using the skeletal model, little is known about its histology. In addition, do mice really have the AP?.

This study clarified fetal developmental changes in the sphenoid in order to determine developmental processes of the AP in humans. In addition, we reconsidered the definition of the AP by comparing humans and rats with mice. We also investigated the boundary between the AP and the AT in humans, mice, and rats.

## Materials and methods

### Sample preparation

This study was conducted in compliance with the provisions of the Helsinki Declaration. In addition, it was approved by the ethics committee of the Universidad Complutense de Madrid, Spain (B-08/374), and Tokyo Dental College, Japan (No. 932). At the time of donation, we received a written consent for the research from their parents.

We used 20 embryos and fetuses from the collection at the Embryology Institute of the Universidad Complutense de Madrid (Table 1). The greatest length (GL) of the embryos was 21–28 mm (Carnegie Stages [CS] 20–23; 7–8 WD). The GL of the fetuses was 37–276 mm (9–32 WD). We used the GL and external and internal criteria to determine the postconceptional age, as previously described [17].

**Table 1:** Details of the embryonic periods.

Carnegie stage or Weeks	CR length(mm)	Embryo or Fatus	Results
20	21	NO2	Fig 6A
20	21	BOT	
20	21	E1	Figs 1A–1C and 7A
22	26	C1	
22	27	Mes2	
23	28	BR4	Figs 1G–1I and 6B
9 weeks	37	Me1	Figs 2C–2F, 5D and 7B
9 weeks	38	O1	
10 weeks	46	JR6	
11 weeks	52	CA6	
12 weeks	62	B403	
12 weeks	74	HL30	
12 weeks	75	PT	Figs 3C–3G and 4C–4E
14 weeks	100	B6	Figs 6C and 7C
15 weeks	120	GEO	
16 weeks	131	CU2	
19 weeks	150	B28	
28 weeks	228	POI	
31 weeks	274	C34	Fig 6D
32 weeks	276	OA	

All specimens were the products of ectopic pregnancies or spontaneous abortions. They were fixed in 10% neutral formalin and then embedded in paraffin for further processing. Next, 10–25-μm-thick histological sections were cut and stained with hematoxylin and eosin (H & E), azan, orange-fuchsin, and Bielschowsky’s silver stain. For 3D reconstruction, we loaded digital images of the serial sections into Amira (Visage Imaging, Inc.) using a voxel size appropriate for the section thickness.

To determine whether the AP is a common structure in mammals, we used 20 C57BL6J mice from embryonic days (E) 12–18 and 4 Wister rats form E17 and E20. The experiments were approved by the IACUC Committee at Tokyo Dental College. The presence of vaginal plugs indicated pregnancy, and the date of detection was designated as E0.5. Timed-pregnant females (E12-E18 in mice, and E17 and E20 in rats) were euthanized by CO_2_ inhalation. The gravid uterus was extracted and suspended in a bath of cold 10% phosphate-buffered saline, and embryos were harvested after amnionectomy and removal of the placenta. The embryos were fixed in 4% phosphate-buffered paraformaldehyde, followed by demineralization using 10% ethylenediaminetetraacetic acid. Next, the specimens were embedded in paraffin blocks and cut into a series of 5- and 10-μm-thick frontal histological sections using a sliding microtome. Finally, the sections were stained using H & E and toluidine blue.

### Sphenoid morphology

We used five adult C57BL6J mice for observation of osseous morphology. Imaging was performed using a micro–computed tomography system (HMX 225Actis4; Tesco Co., Tokyo, Japan) under the following conditions: tube voltage, 100 kV; tube current, 120 μA; slice width, 50 μm; matrix size, 512 × 512; and slice voxel size, 52.7 × 52.7 × 50 μm. We used images of the histological slides to reconstruct 3D images in VGStudio 3D reconstruction software (Volume Graphics, Heidelberg, Germany).

## Results

### Sphenoid development at 7 and 8 WD

**Fig 1:**
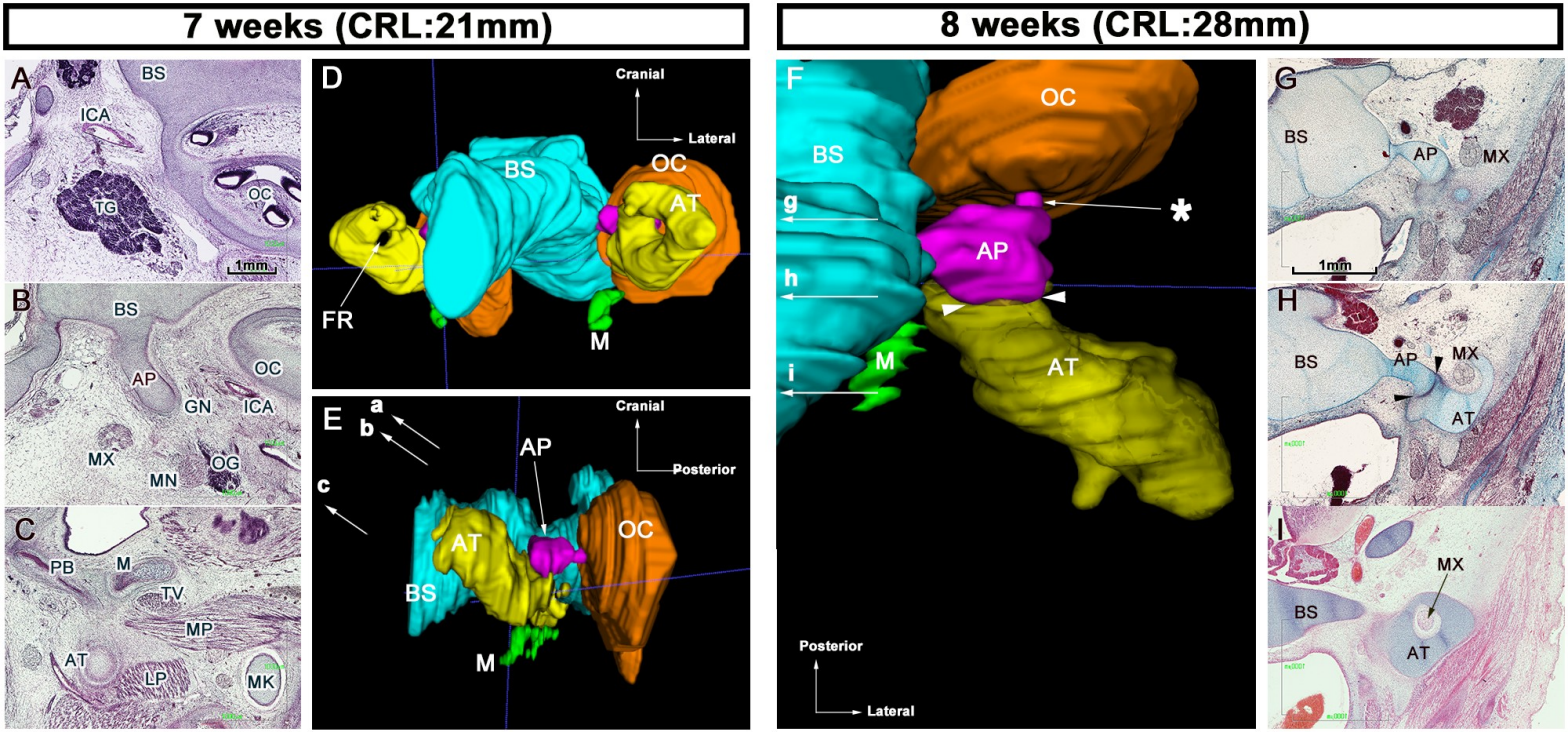
Sphenoid development at 7 and 8 WD. (A-C) Transverse sections at 7 WD. The AP was continuous with the basisphenoid. (D and E) 3D reconstructions at 7 WD. (F) 3D reconstruction at 8 WD. (D) Anterior view, (E) lateral view, and (F) superior view. (G–I) Frontal sections at 8 WD. Arrows (a–c) in (E) correspond to sections in (A–C), respectively. Arrows (g–i) in (F) correspond to sections in (G–I), respectively. (A–C) and (G–H) are obtained at the same magnification. Color code: blue, BS; pink, AP; yellow, AT; green, M; orange, OC. AP, alar process; AT, ala temporalis; BS, basisphenoid; GN, greater petrosal nerve; ICA, internal carotid artery; LP, lateral pterygoid muscle; M, medial pterygoid process; MK, Meckel’s cartilage; MN, mandibular nerve; MX, maxillary nerve; OC, otic capsule; OG, otic ganglion; PB, palatine bone; TG, trigeminal ganglion; TV, tensor veli palatine; WD, weeks of development.

The body and GW of the fetal sphenoid comprised the basisphenoid, AP, and AT, which are made of cartilaginous tissue (Fig 1). The AP was continuous with the basisphenoid (Fig 1B and 1H) but was separated from the AT by a thick perichondrium (Fig 1F and 1H, arrowheads), and its posterior end was in contact with the otic capsule (OC) (Fig 1F, asterisk). We also observed a hole, the future foramen rotundum, in the AT cartilage (Fig 1D and 1I), through which the maxillary nerve passes (Fig 1H and 1I). In addition, the medial pterygoid process was inferior to the AT (Fig 1C–1E).

### Sphenoid development at 10 WD

**Fig 2:**
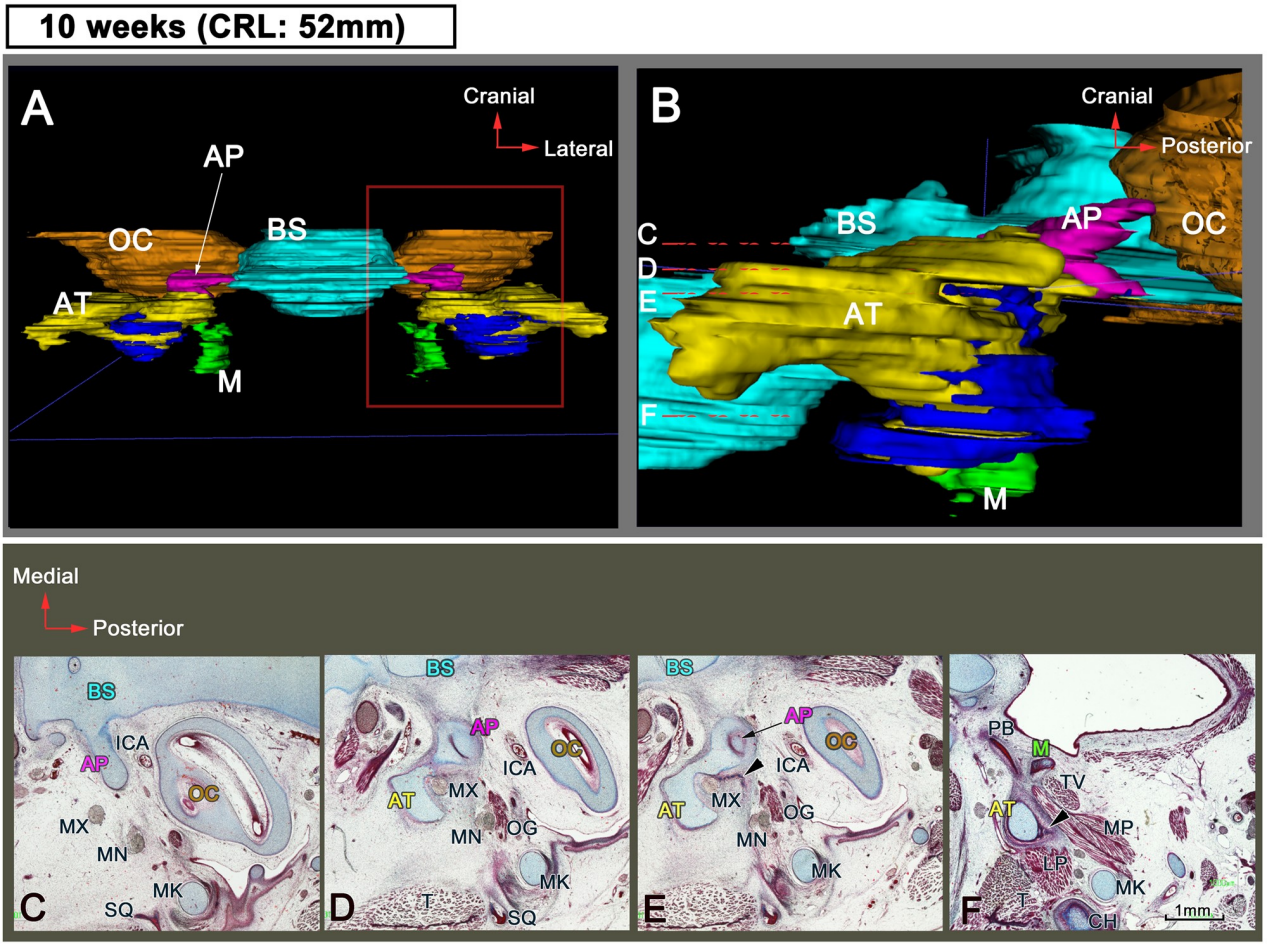
Sphenoid development at 10 WD. (A and B) 3D reconstructions and (C–F) transverse sections. (A) Anterior view and (B) lateral view. Dotted lines (c–f) in (B) correspond to sections in (C–F), respectively. (E and F) Membranous bones around the AT (arrowheads). (C–F) are obtained at the same magnification. Color code: blue, BS; pink, AP; yellow, AT; green, M; orange, OC; dark blue, membranous bones. AP, alar process; AT, ala temporalis; BS, basisphenoid; ICA, internal carotid artery; LP, lateral pterygoid muscle; M, medial pterygoid process; MK, Meckel’s cartilage; MN, mandibular nerve; MX, maxillary nerve; OC, otic capsule; OG, otic ganglion; PB, palatine bone; SQ, squamosal bone; TG, trigeminal ganglion; T, temporalis muscle or temporal bone; TV, tensor veli palatine; WD, weeks of development.

The AP was located between the AT and the basisphenoid (Fig 2A–2D), and a part of it was surrounded by the AT (Fig 2C and 2E). Its posterior end extended compared to the previous stage (Fig 2B). A few membranous bones (Fig 2E and 2F; arrow head) also appeared around the AT (Fig 2A and 2B). The squamosal part of the temporal bone was first identified laterally to Meckel’s cartilage (Fig 2C–2F). In addition, the maxillary nerve traveled through the AT (Fig 2D and 2E).

### Sphenoid development at 12 WD

**Fig 3:**
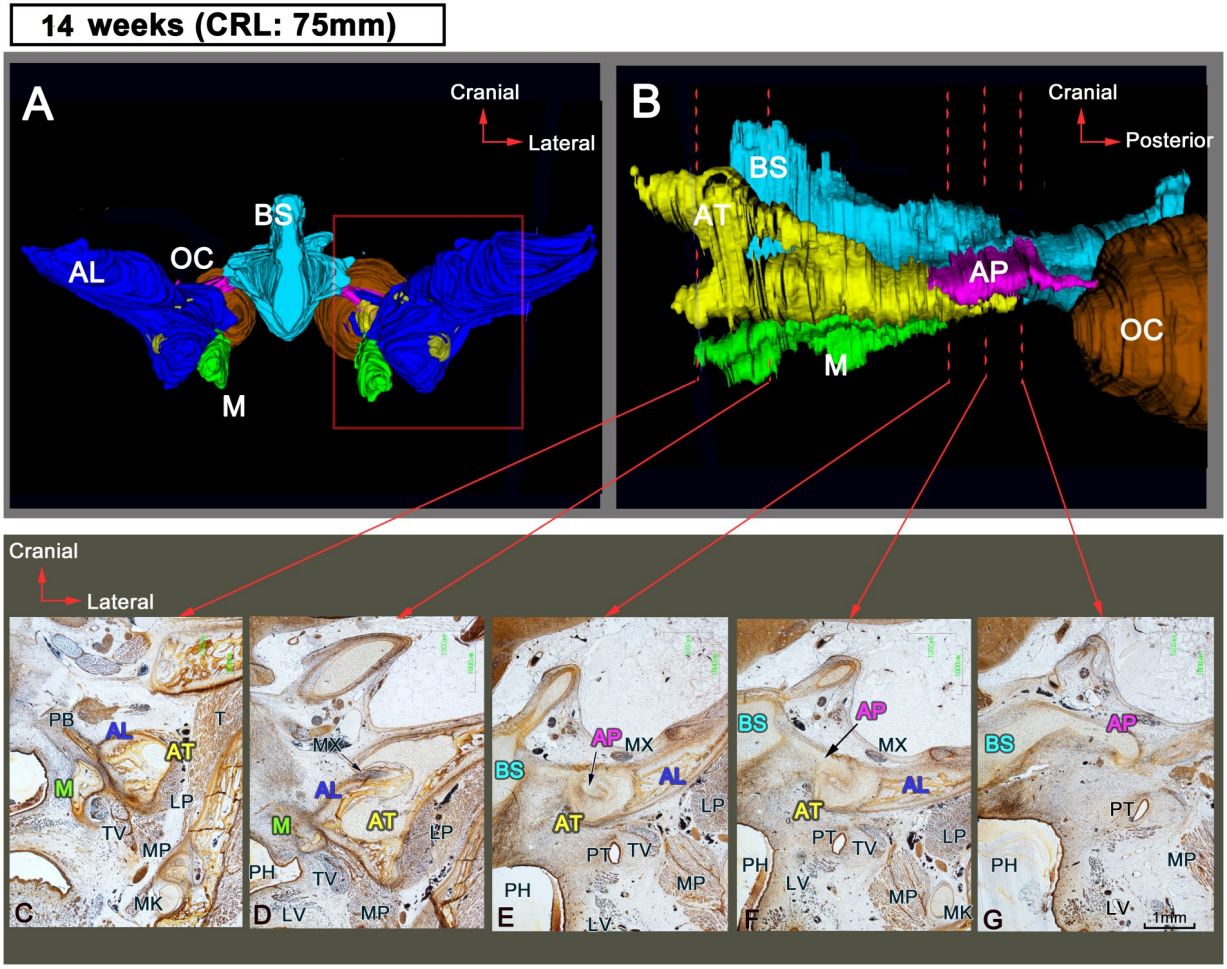
Sphenoid development at 14 WD. (A and B) 3D reconstructions and (C–G) frontal sections. (A) Anterior view and (B) lateral view. (C–G) are obtained at the same magnification. Color code: blue, BS; pink, AP; yellow, AT; green, M; orange, OC; dark blue, AL (membranous bones). AL, alisphenoid; AP, alar process; AT, ala temporalis; BS, basisphenoid; LP, lateral pterygoid muscle; LV, levator veli palatini; M, medial pterygoid process; MK, Meckel’s cartilage; MX, maxillary nerve; OC, otic capsule; PB, palatine bone; PH, primitive pharynx; PT, pharyngotympanic tube; T, temporalis muscle or temporal bone; TV, tensor veli palatine; WD, weeks of development.

**Fig 4:**
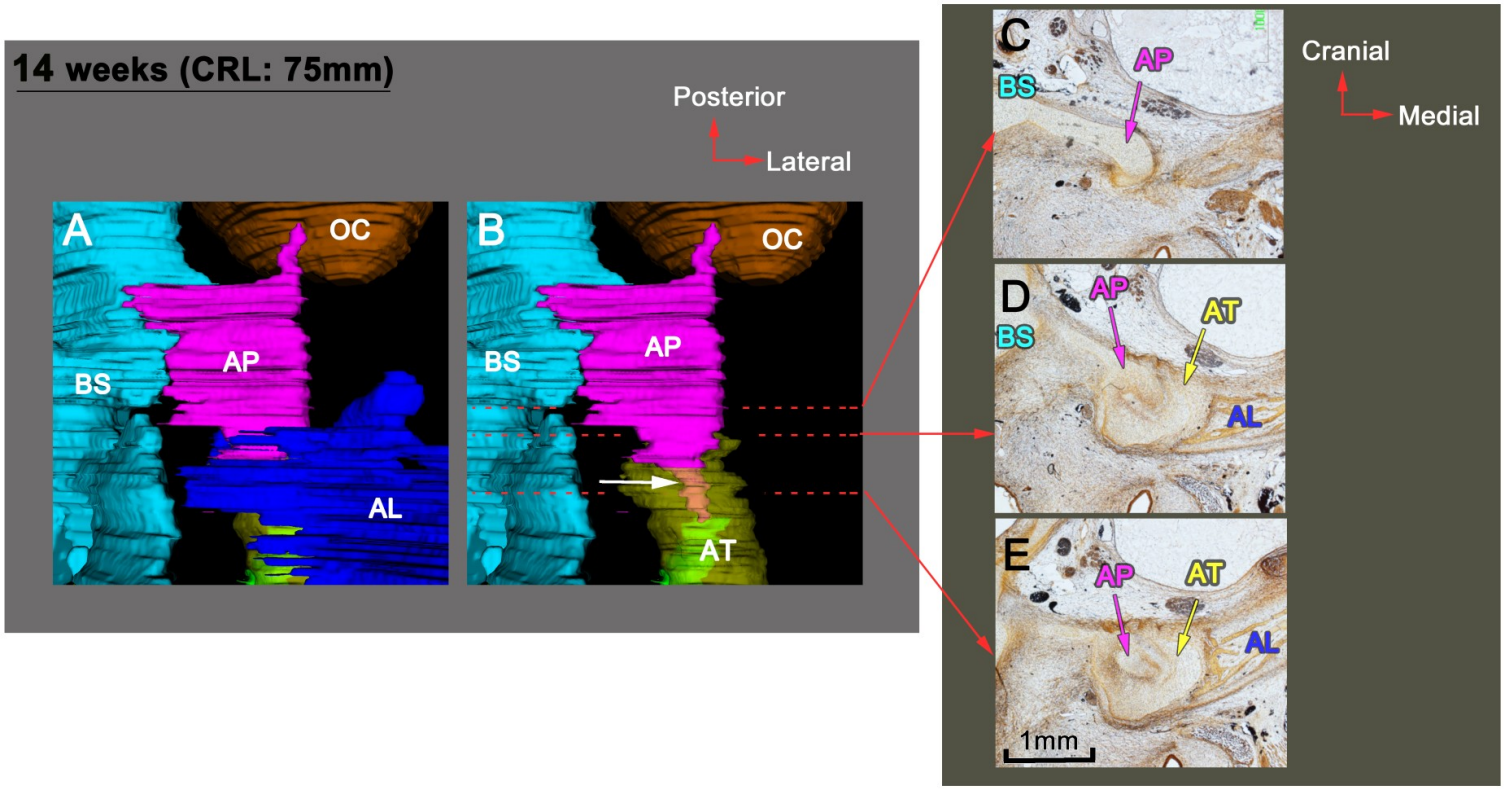
A-A connection at 14 WD. (A and B) 3D reconstructions. (A) Superior view and (B) superior view excluding membranous bones. (C–E) Frontal sections. The AP and the AT are connected by a key-and-keyhole structure indicated with an arrow in (B). (C–E) are obtained at the same magnification. Color code: blue, BS; pink, AP; yellow, AT; orange, OC; dark blue, AL (membranous bones). AL, alisphenoid; AP, alar process; AT, ala temporalis; BS, basisphenoid; OC, otic capsule; WD, weeks of development.

The AT was surrounded by membranous bones (Fig 3). The AP and the AT were connected by a key-and-keyhole structure because the AP extended anteriorly compared to the previous stage (Fig 4). In addition, the maxillary nerve passed along the GW (Fig 3E and 3F) and then perforated the newly formed foramen rotundum (Fig 3D) composed of membranous bone.

### Difference between human and mouse

**Fig 5:**
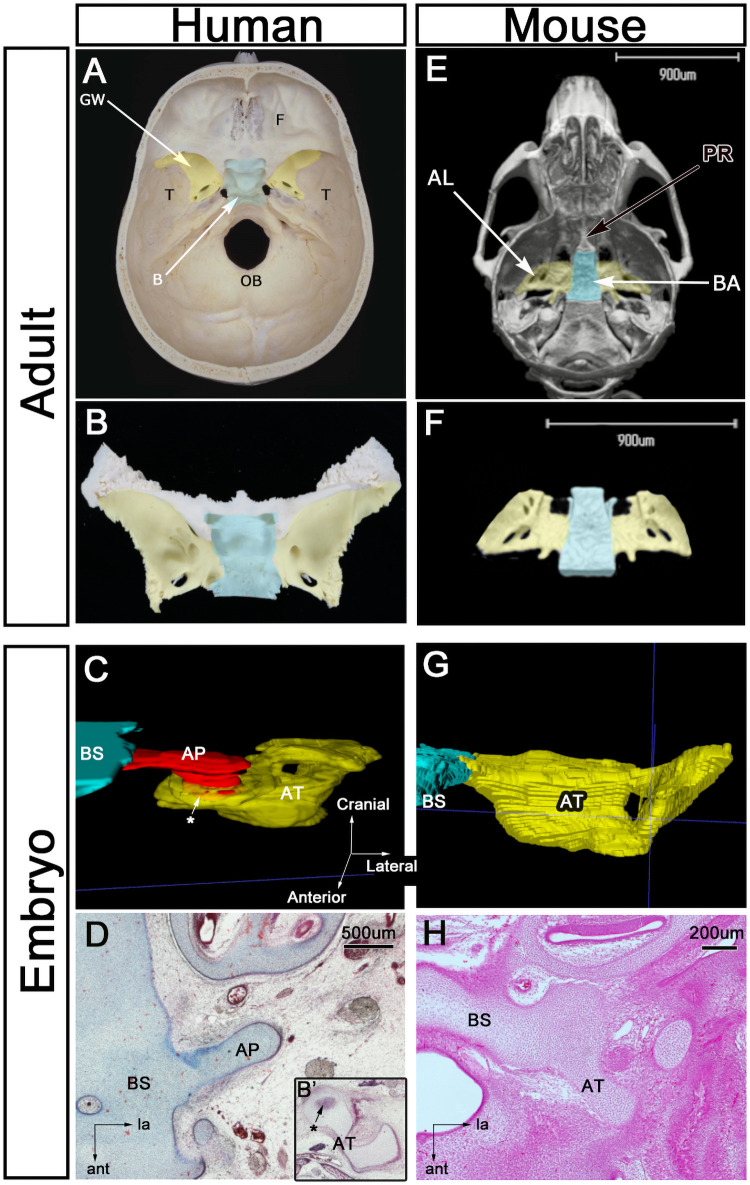
Differences between human and mice skulls. (A–D) Human skulls and (E–H) mouse skulls. (A and B) Sphenoid bone in the adult human. (C and D) 3D reconstructions of the transverse section of the fetal sphenoid at 10 WD, showing a temporary structure connecting the AT to the basisphenoid. (E and F) 3D reconstructions obtained using micro–computed tomography. (G and H) 3D reconstructions of the transverse section of the mouse sphenoid at E 14. The AP is absent. Color code: blue, B or BS; red, AP; yellow, AT or GW of the AT. AP, alar process; AT, ala temporalis; B, body of the sphenoid; BS, basisphenoid; F, frontal bone; GW, greater wing of the sphenoid; ICA, internal carotid artery; OB, occipital bone; PR, presphenoid; E, embryonic day.

The sphenoid in the adult human skull was similar to that of the adult mouse (Fig 5A, 5B, 5E and 5F). However, its components showed interspecific differences during fetal development (Fig 5C, 5D, 5G and 5H). In the human fetus, the AP was a temporary structure connecting the AT (anlage of the GW) to the basisphenoid (anlage of the body; Fig 5C). Although the AP is located between the AT and the basisphenoid in mammals [12,18], we showed histologically that there is no boundary between the AT and the basisphenoid in mice (Fig 5G and 5H), indicating that the AP is absent in mice (Fig 5).

**Fig 6:**
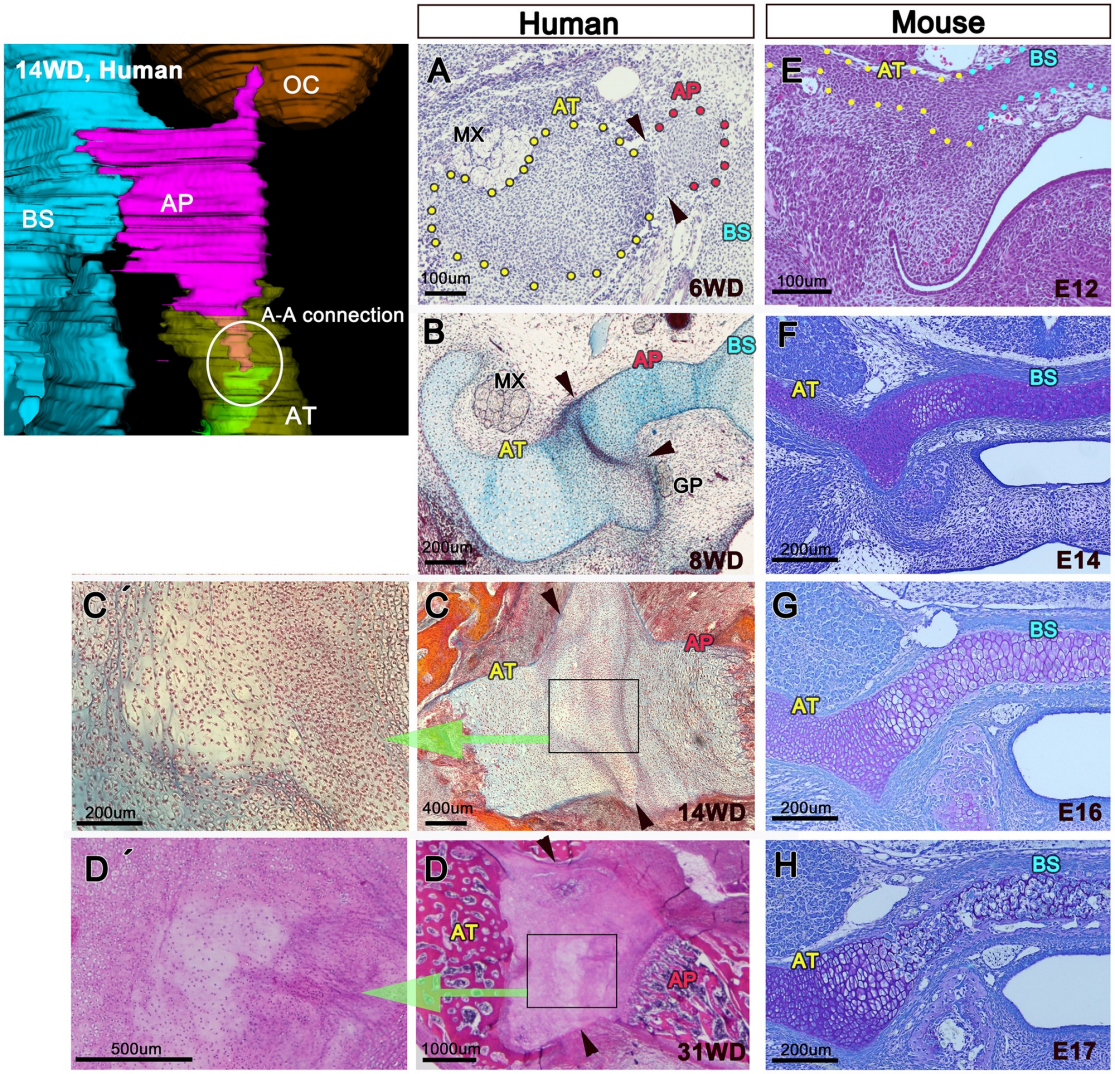
A-A connection in humans and mice. (A–D) A-A connection in humans (arrowheads). (C’ and D’) Enlargement of (C and D), respectively. (E–H) Middle cranial base in mice. Histological analysis in humans showed an unusual structure (A-A connection) between the AT and the AP. Mice do not have the A-A connection. AP, alar process; AT, ala temporalis; BS, basisphenoid; GP, greater petrosal nerve; MX, maxillary nerve; OC, otic capsule.

In addition, we observed an unusual structure between the AT and the AP in the human fetus. Although this structure resembled an immature joint, no cavitation developed (Fig 6A–6D). We named this structure the “A-A connection”. In the mouse embryo, however, the AT was directly connected with the basisphenoid (Fig 6E–6H).

### Temporary continuity between the posterior end of the alar process and the otic capsule

**Fig 7:**
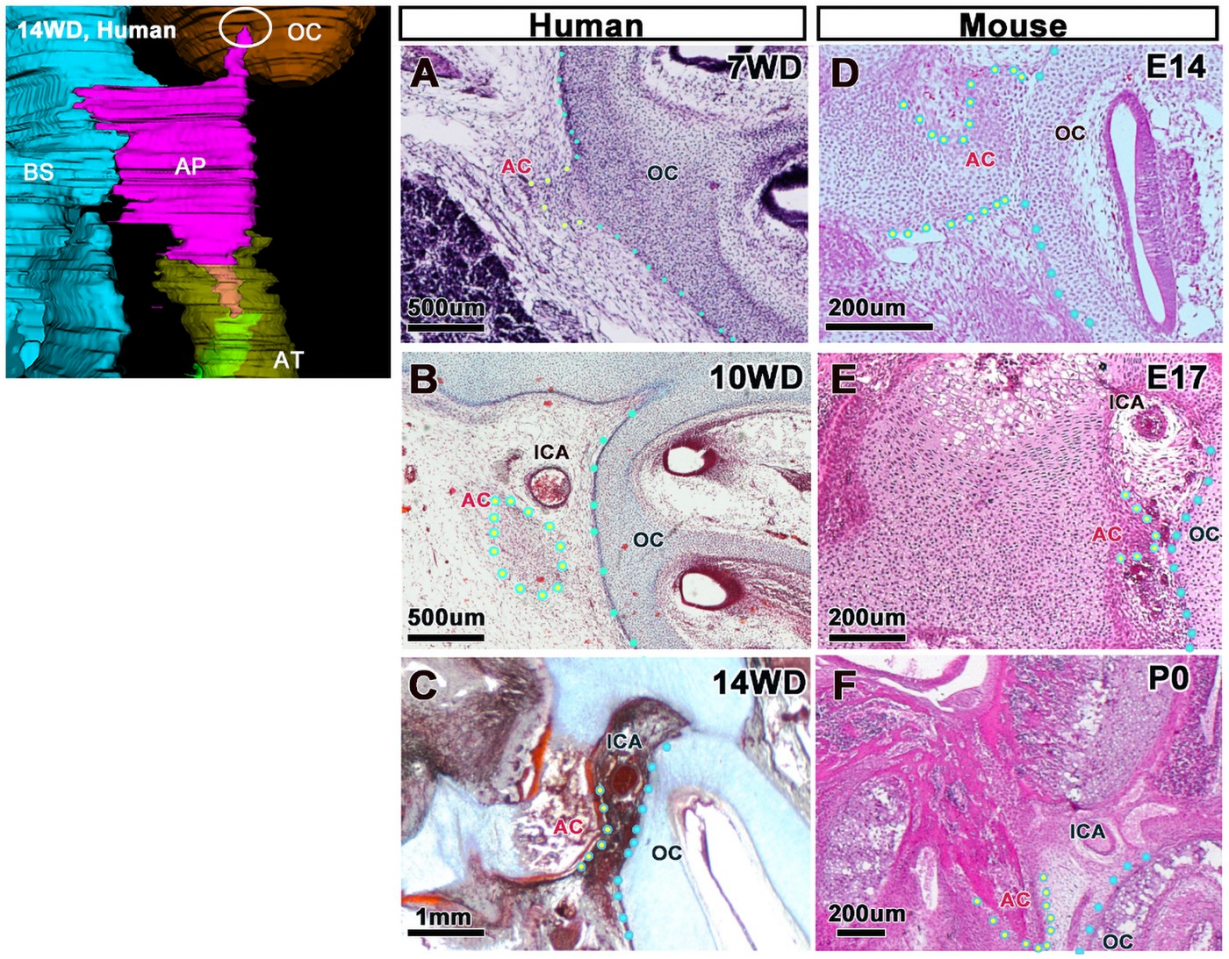
Temporary continuity between the posterior end of the AP and the OC. (A–C) Posterior end of the AP and the OC in humans. (D–F) Posterior end of the AP and the OC in mice. The timing of separation of the AP from the OC differs between humans and mice. AP, alar process; AT, ala temporalis; BS, basisphenoid; ICA, internal carotid artery; OC, otic capsule.

At 7 WD, we found a temporary contact between the posterior end of the AP and the OC (Fig 7A). However, these two components separated with sphenoid bone development (Fig 7B and 7C). To determine whether this cartilaginous continuity is a common structure in mammals, we studied mouse embryos. We found that the end of the AP is in contact with the OC during the fetal period (Fig 7D) but separates right before birth (E17; Fig 7E and 7F).

### A-A connection in rats

**Fig 8:**
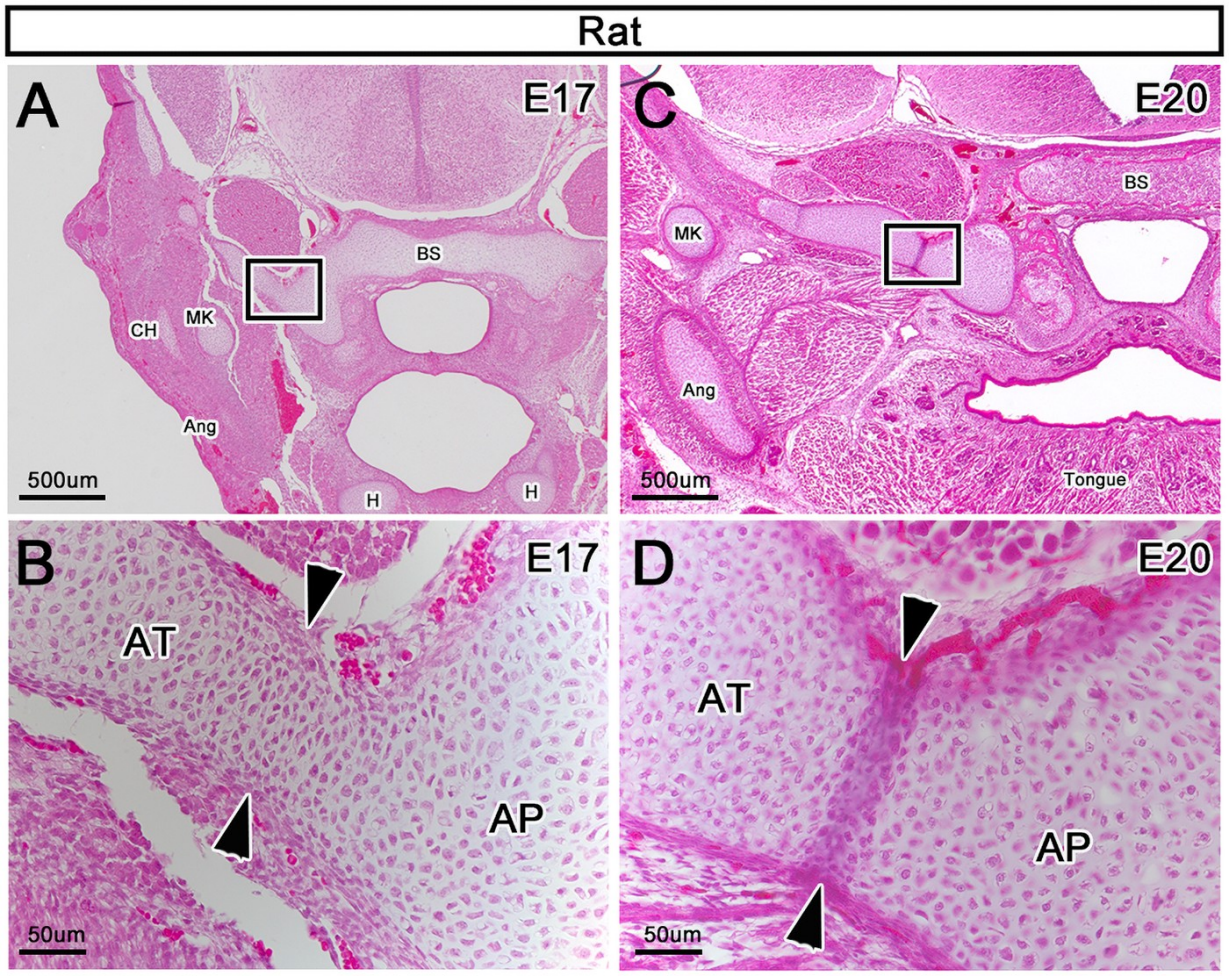
A-A connection in rats. (A and B) Frontal sections at E17. (C and D) Frontal sections at E20. (B and D) Enlargement of (A and C), respectively. (B) The AP is continuous with the AT at E17 (arrowheads). (D) The AP is separated from the AT by a thick perichondrium at E20 (arrowheads). Ang, angular process; AP, alar process; AT, ala temporalis; BS, basisphenoid; CH, condylar head; H, hyoid bone; MK, Meckel’s cartilage.

The AP was continuous with the AT at E17 (Fig 8A and 8B), but was separated from the AT by a thick perichondrium at E20 (Fig 8C and 8D). Therefore, we also found the “A-A connection” in rats, similar to humans.

## Discussion

The sphenoid has a cartilaginous connecting apparatus, the AP, during the fetal period [11], but little is known about its development. This study analyzed the development of the AP in humans, mice, and rats. In humans, the AP was continuous with the basisphenoid but was separated from the AT by a thick perichondrium. Then, the AP–AT connection had a key-and-keyhole structure. Unlike a joint, no cavitation developed in this connection. In mice, however, the AT is directly continuous with the basisphenoid, and there is no boundary between the two during the fetal period. In rats, we found the AP was separated from the AT by a thick perichondrium during the fetal periods. A previous study [6] has shown that no boundary appears between the basisphenoid and the AT in mice. Therefore, the AP is not included in components of the mouse sphenoid. However, the definition of the AP is unclear. Earlier, authors probably called the structure connecting the AT to the OC as the AP. We defined the alar process as follows: the AP is temporally separated from the AT by a thick perichondrium or a key-and-keyhole structure during the fetal period (Figs 4, 6 and 8). Further studies are required in order determine the presence or absence of the AP in other mammalian species.

The human brain rapidly increases in size during fetal development. Its volume increases 164.4-fold from CS 13–23 [19–21]. During the fetal period, the peak increase in brain weight occurs between 9 and 18 weeks [22]. During this period, human fetuses have a large gap between the cerebrum and the calvaria [23–25]. In contrast, the cranial base is close to the brainstem, thalamus, and hypothalamus [23–25]. This study demonstrated the development of a temporary connecting apparatus from 7 to 14 WD. During this period, the AP joins with the AT by a key-and-keyhole structure (the A-A connection). In addition, the posterior end of the AP is in contact with the otic capsule and then separates with development. These findings strongly suggest that the posterior end of the AP separates from the OC as a result of mechanical stress arising from the development of these three structures, except the cerebrum, with the A-A connection acting as a shock absorber to counter the stress. However, the mechanical stress exerted by the brain is difficult to test experimentally. Contrary to this hypothesis, 3H1 Br/Br mice show an inversion mutation affecting the six2/six3 complex, resulting in the absence of the presphenoid and a reduction of the basisphenoid. However, the cochlear complex and brain still appear largely unaffected [26,27].

De Beer [28] and Youssef [15] described the alicochlear commissure, which connects the AT to the OC. In mice, a neural crest–mesoderm boundary is located in the alicochlear commissure [16], which originates from both the neural crest and the mesoderm. The AT is connected to the OC by the alicochlear commissure. In contrast, the alicochlear commissure of bat embryos is an outgrowth of the OC connecting to the AP [25]. In addition, in humans, the posterior end of the AP is the alicochlear commissure. Therefore, the alicochlear commissure of bat embryos seems to derive from the mesoderm, and the origin of the human alicochlear commissure seems to be the neural crest. The origins of the alicochlear commissure seem to differ among animal species.

The vidian nerve formed by the junction of the greater petrosal and deep petrosal nerves passes through the vidian canal [8]. A previous study on human fetuses [12] has shown that the vidian nerve passes inferior to the A-A connection. In addition, the vidian canal consists of the medial pterygoid (medial part), AT (lateral part), and A-A connection (superior part) [12]. Therefore, we believe that the AP contributes to the superior part of the pterygopalatine osseous complex in the sphenoid. In contrast, a small process between the body and GW of the sphenoid is called the sphenoidal ligula [8]. During the fetal period, the AP is located between the basisphenoid and the AT [12], and its posterior end corresponds to the alicochlear commissure. Therefore, the alicochlear commissure might contribute to the sphenoidal ligula.

## Conclusion

The AP can be defined as follows: the AP is temporally separated from the AT by a thick perichondrium or a key-and-keyhole structure during the fetal period (Figs 4, 6 and 8). The AP is absent in mice, and its presence/absence in other mammals needs further investigation.

## Supporting information

S1 Fig Sphenoid development at 14WD. Panels A-C are transverse sections. Abbreviations: AL = alisphenoid; AP = alar process; AT = ala temporalis; BS = basisphenoid; ICA = internal carotid artery; LP = lateral pterygoid muscle; M = medial pterygoid process; Ma = masseter muscle; MP = medial pterygoid process; MK = Meckel’s cartilage; MX = maxillary nerve; L; lateral pterygoid process; OC = otic capsule; P = palatine bone; SQ = squamosal bone; TG = trigeminal ganglion; T = temporalis muscle; TVP = tensor veli palatini.
(TIF)

S2 Fig (JPG)

S3 Fig (JPG)
