# Supplementary figures and images for: Development of the cartilaginous connecting apparatuses in the fetal sphenoid, with a focus on the alar process
Source: PLoS One. 2021 Jul 12;16(7):e0251068. doi: 10.1371/journal.pone.0251068 (PMC8274926; doi:10.1371/journal.pone.0251068)

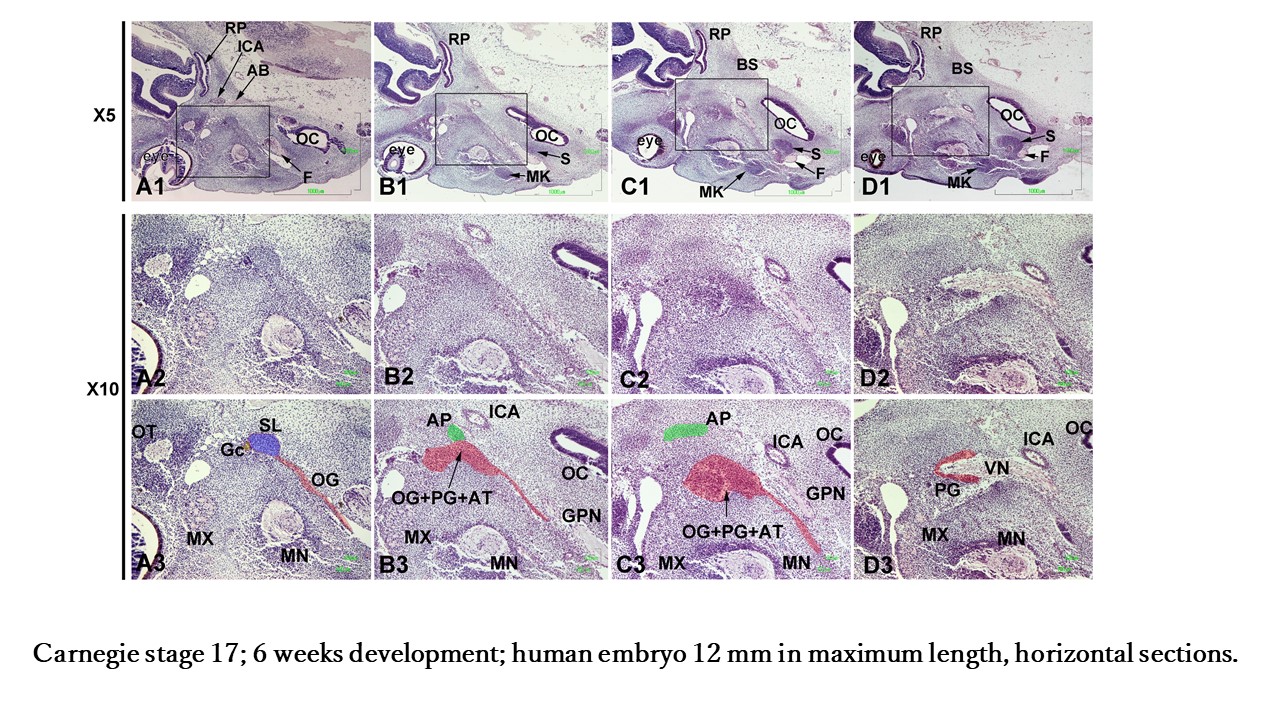

Supplement: S2 Fig — (JPG) [file pone.0251068.s002.jpg]

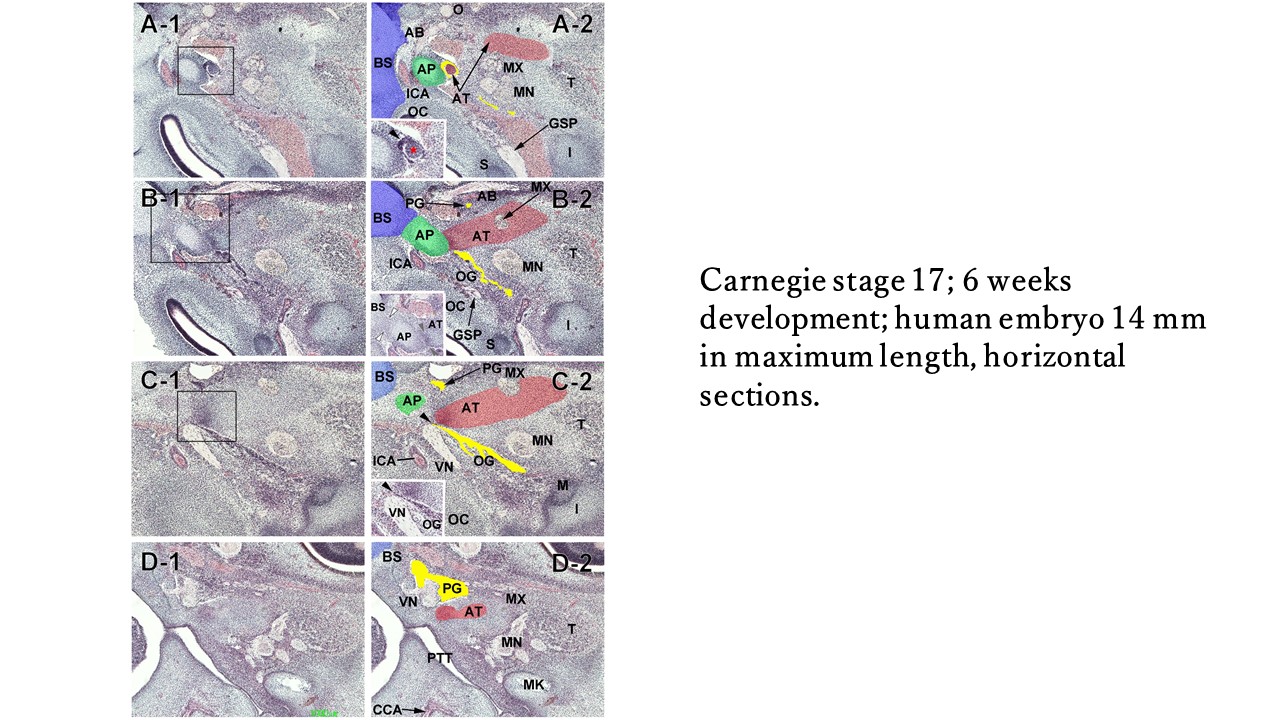

Supplement: S3 Fig — (JPG) [file pone.0251068.s003.jpg]
